# Calibration of BeiDou Triple-Frequency Receiver-Related Pseudorange Biases and Their Application in BDS Precise Positioning and Ambiguity Resolution

**DOI:** 10.3390/s19163500

**Published:** 2019-08-10

**Authors:** Fu Zheng, Xiaopeng Gong, Yidong Lou, Shengfeng Gu, Guifei Jing, Chuang Shi

**Affiliations:** 1 School of Electronic and Information Engineering, Beihang University, 37 Xueyuan Road, Beijing 100083, China; 2 GNSS Research Center, Wuhan University, Luoyu Road 129, Wuhan 430079, China; 3 BeiDou Belt and Road School, Beihang University, 37 Xueyuan Road, Beijing 100083, China

**Keywords:** BDS, pseudorange bias, triple-frequency, single point positioning, ambiguity resolution

## Abstract

Global Navigation Satellite System pseudorange biases are of great importance for precise positioning, timing and ionospheric modeling. The existence of BeiDou Navigation Satellite System (BDS) receiver-related pseudorange biases will lead to the loss of precision in the BDS satellite clock, differential code bias estimation, and other precise applications, especially when inhomogeneous receivers are used. In order to improve the performance of BDS precise applications, two ionosphere-free and geometry-free combinations and ionosphere-free pseudorange residuals are proposed to calibrate the raw receiver-related pseudorange biases of BDS on each frequency. Then, the BDS triple-frequency receiver-related pseudorange biases of seven different manufacturers and twelve receiver models are calibrated. Finally, the effects of receiver-related pseudorange bias are analyzed by BDS single-frequency single point positioning (SPP), single- and dual-frequency precise point positioning (PPP), wide-lane uncalibrated phase delay (UPD) estimation, and ambiguity resolution, respectively. The results show that the BDS SPP performance can be significantly improved by correcting the receiver-related pseudorange biases and the accuracy improvement is about 20% on average. Moreover, the accuracy of single- and dual-frequency PPP is improved mainly due to a faster convergence when the receiver-related pseudorange biases are corrected. On the other hand, the consistency of wide-lane UPD among different stations is improved significantly and the standard deviation of wide-lane UPD residuals is decreased from 0.195 to 0.061 cycles. The average success rate of wide-lane ambiguity resolution is improved about 42.10%.

## 1. Introduction

With the development of the Global Navigation Satellite System (GNSS), multi-frequency and multi-GNSS are the main characteristics of future satellite navigation. Multiple GNSS systems provide abundant observations for precise positioning, atmospheric modeling, and many other applications. However, it also brings great challenges for GNSS precise data processing when multi-frequency and multi-GNSS measurements are combined [1]. For instance, code biases will affect the convergence performance of precise point positioning (PPP), the extraction of total electron content (TEC) in ionospheric modeling, the ability of ambiguities resolution in PPP, and relative carrier phase positioning when multi-frequency GNSS measurements are used [2].

The topic of GNSS hardware biases has received a lot of attention in recent years, including satellite-related/receiver-related and code-related/phase-related biases of different GNSS systems [3,4,5,6]. As a new emerging satellite system, the study of BDS hardware biases also attracts the GNSS community’s interests, including BDS differential code bias (DCB) [7,8,9], inter-satellite-type bias (ISTB), which is a receiver-dependent bias between BDS different satellite types [10], and BDS group delay variations, which are elevation-dependent code bias variations [11,12]. 

Except GLObal NAvigation Satellite System (GLONASS), it is always assumed that the receiver hardware biases are the same for satellites belonging to the same GNSS system, which uses code division multiple access (CDMA). Correspondingly, in the satellite clock estimation with GNSS network, the general processing strategy is to estimate one receiver clock per station, which contains receiver hardware delay, and one satellite clock per satellite. However, this is not practical. Based on the data from different types of receivers, reference [13] analyzed the characteristics of BDS receiver pseudorange biases and found that the receiver bias of each BDS satellite is not identical, which resulted in 1.5 ns clock bias in the BDS satellite clock estimation with inhomogeneous receivers. It means the characteristics of pseudorange biases must be carefully studied and processed, or the accuracy of GNSS data processing results will be degraded, such as satellite clock and DCB estimation.

The problem of pseudorange biases has received great attention since the early days of Global Positioning System (GPS). The study has been triggered by signal anomaly of the GPS satellites Space Vehicle Number (SVN) 19 and SVN 49. It is demonstrated that the signal anomaly of SVN 19 and SVN 49 is caused by signal distortions and reflections of GPS satellites [14]. The signal anomaly of SVN 19 occurred in March 1993 and led to significant degradations of differential GPS positioning results with mixed receivers, and the signal anomaly of SVN 49 caused a secondary-path signal with a delay of approximately 30 nanoseconds, which has the appearance of a multipath error [14,15]. Additionally, the correlator design and the multipath mitigation techniques of different receivers for signal distortions are studied in many publications [16,17,18]. With the measurements from geodetic receivers, reference [19] studied the dependency of pseudorange biases on correlator design and pointed that the signal distortions differ from satellite to satellite, the receiver’s filter response differs for each satellite and causes satellite-dependent biases, which will affect the positioning and timing accuracy.

By studying the characteristics of BDS pseudorange biases of different types of receivers, it is found that the BDS pseudorange biases are related to receiver manufacturers and receiver models, and they can reach up to about 3 ns among different types of receivers [13]. In order to avoid the effect of ionospheric delay, the BDS receiver-related pseudorange biases are calibrated by using B1/B2 ionosphere-free (IF) combination. It is demonstrated that the initial bias of satellite clock estimation and positioning accuracy of dual-frequency single point positioning (SPP) are significantly improved when the BDS pseudorange biases are corrected. However, this correction model is only suitable for data processing of B1/B2 IF combination and is not applicable in BDS single-frequency or triple-frequency data processing.

In order to further improve the performance of BDS precise data processing, it is essential to deeply study the characteristics of BDS signal delay biases. In this contribution, BDS receiver-related pseudorange biases of triple-frequency observations will be calibrated and validated. The observation model and method for receiver-related pseudorange bias calibration will be introduced in the second section. In the section on validation of BDS pseudorange biases, the bias corrections will be validated by BDS SPP, wide-lane uncalibrated phase delays (UPD), and ambiguity resolution. Finally, some conclusions and suggestions will be presented.

## 2. Observation Model

In GNSS data processing, hardware delays are commonly partitioned into a sum of satellite- and receiver-specific biases. However, it is revealed that satellite-plus-receiver hardware delay cannot be rigorously split into a sum of two independent parts, there are pseudorange biases related to different receiver models or types, which are different between satellites at the receiver-end [19]. Thus, considering the receiver–satellite pair hardware delay, the raw GNSS pseudorange observation is defined as follows:

(1) $$\left\{ \begin{array}{l} P_{r,f}^{s}=\rho +c\cdot (t_{r}+b_{r,f})-c\cdot (t^{s}+b_{f}^{s})+c\cdot b_{type(r),f}^{s}+\alpha^{s}\cdot T_{z}+\beta_{f}\cdot I_{r}^{s}+\varepsilon_{P_{r,f}}\\ \Phi_{r,f}^{s}=\rho +c\cdot (t_{r}+B_{r,f})-c\cdot (t^{s}+B_{f}^{s})+\alpha^{s}\cdot T_{z}-\beta_{f}\cdot I_{r}^{s}+\lambda_{f}\cdot N_{f}+\varepsilon_{\Phi_{r,f}} \end{array}\right.$$

where $P_{r,\;f}^{s}$ and $\Phi_{r,\;f}^{s}$ represent the pseudorange and phase measurements on frequency *f* (*f* = 1, 2, 3) from receiver *r* to satellite *s* (*s* = 1, 2, …, *m*), *m* is the number of satellites tracked by receiver *r*; ρ is the geometric distance with antenna phase center corrections; *c* denotes the speed of radio waves in vacuum; *t_r_* and *t^s^* are receiver and satellite clock error, respectively; *T*_z_ is the zenith tropospheric delay that can be converted to slant with the mapping function *α^s^*; $b_{r,f}$ and $b_{f}^{s}$ are the receiver-specific and satellite-specific pseudorange hardware delay, respectively; $b_{\mathrm{type}(r),f}^{s}$ is the receiver–satellite pair hardware delay and related to receiver type; $B_{r,f}$ and $B_{f}^{s}$ are the receiver-specific and satellite-specific phase hardware delay, respectively; $I_{r}^{s}$ denotes the line-of-sight total electron content with the frequency-dependent factor $\beta_{f}$; $\lambda_{f}$ and $N_{f}$ are wavelength and integer ambiguity of phase observation; $\varepsilon_{P_{r,\;f}}$ and $\varepsilon_{\Phi_{r,\;f}}$ represents observation noise of pseudorange and phase. 

The B1/B2 IF combination of receiver-related pseudorange biases are given and validated [13]. In this paper, to derive the original receiver-related pseudorange biases of BDS, two ionosphere-free and geometry-free (IFGF) combinations are adopted:

(2) $$\left\{ \begin{array}{l} {\mathrm{MW}}_{r}^{s}=\frac{\left(f_{1}\cdot P_{r,1}^{s}+f_{2}\cdot P_{r,2}^{s}\right)}{(f_{1}+f_{2})\cdot \lambda_{1,-1,0}}-(\frac{\Phi_{r,1}^{s}}{\lambda_{1}}-\frac{\Phi_{r,2}^{s}}{\lambda_{2}})\\ P_{r,IFGF}^{s}=(\frac{f_{1}^{2}}{f_{1}^{2}-f_{2}^{2}}-\frac{f_{1}^{2}}{f_{1}^{2}-f_{3}^{2}})\cdot P_{r,1}^{s}-\frac{f_{2}^{2}}{f_{1}^{2}-f_{2}^{2}}\cdot P_{r,2}^{s}+\frac{f_{3}^{2}}{f_{1}^{2}-f_{3}^{2}}\cdot P_{r,3}^{s} \end{array}\right.$$

where ${\mathrm{MW}}_{r}^{s}$ is Melbourne-Wübbena (MW) combination [20,21] and $P_{r,IFGF}^{s}$ is IFGF combination of triple-frequency pseudorange; $\lambda_{1,-1,0}$ is the wavelength of wide-lane ambiguity. Based on Equation (2), single difference between different receivers is adopted to eliminate the satellite-specific biases:

(3) $$\left\{ \begin{array}{ll} \Delta {\mathrm{MW}}_{r}^{s} & =\frac{\left(f_{1}\cdot \Delta P_{r,1}^{s}+f_{2}\cdot \Delta P_{r,2}^{s}\right)}{(f_{1}+f_{2})\cdot \lambda_{1,-1,0}}-(\frac{\Delta \Phi_{r,1}^{s}}{\lambda_{1}}-\frac{\Delta \Phi_{r,2}^{s}}{\lambda_{2}})\\ & =c\cdot \frac{f_{1}\cdot (\Delta b_{r,1}+\Delta b_{type(r),1}^{s})+f_{2}\cdot (\Delta b_{r,2}+\Delta b_{type(r),2}^{s})}{(f_{1}+f_{2})\cdot \lambda_{1,-1,0}}+(\Delta N_{1}-\Delta N_{2})+c\cdot (\Delta B_{r,1}-\Delta B_{r,2})\\ & =c\cdot \frac{f_{1}\cdot \Delta b_{type(r),1}^{s}+f_{2}\cdot \Delta b_{type(r),2}^{s}}{(f_{1}+f_{2})\cdot \lambda_{1,-1,0}}+(\Delta N_{1}-\Delta N_{2})+c\cdot bias_{r,\mathrm{MW}}\\ \Delta P_{r,IFGF}^{s} & =(\frac{f_{1}^{2}}{f_{1}^{2}-f_{2}^{2}}-\frac{f_{1}^{2}}{f_{1}^{2}-f_{3}^{2}})\cdot \Delta P_{r,1}^{s}-\frac{f_{2}^{2}}{f_{1}^{2}-f_{2}^{2}}\cdot \Delta P_{r,2}^{s}+\frac{f_{3}^{2}}{f_{1}^{2}-f_{3}^{2}}\cdot \Delta P_{r,3}^{s}\\ & =(\frac{c\cdot f_{1}^{2}}{f_{1}^{2}-f_{2}^{2}}-\frac{c\cdot f_{1}^{2}}{f_{1}^{2}-f_{3}^{2}})\cdot (\Delta b_{r,1}+\Delta b_{type(r),1}^{s})-\frac{c\cdot f_{2}^{2}}{f_{1}^{2}-f_{2}^{2}}\cdot (\Delta b_{r,2}+\Delta b_{type(r),2}^{s})+\frac{c\cdot f_{3}^{2}}{f_{1}^{2}-f_{3}^{2}}\cdot (\Delta b_{r,3}+\Delta b_{type(r),3}^{s})\\ & =c\cdot ((\frac{f_{1}^{2}}{f_{1}^{2}-f_{2}^{2}}-\frac{f_{1}^{2}}{f_{1}^{2}-f_{3}^{2}})\cdot \Delta b_{type(r),1}^{s}-\frac{f_{2}^{2}}{f_{1}^{2}-f_{2}^{2}}\cdot \Delta b_{type(r),2}^{s}+\frac{f_{3}^{2}}{f_{1}^{2}-f_{3}^{2}}\cdot \Delta b_{type(r),3}^{s}+bias_{r,P_{IFGF}}) \end{array}\right.$$

where Δ is a single-difference operator between different receivers, e.g., $\Delta P_{r,1}^{s}=P_{r_{1},1}^{s}-P_{r_{2},1}^{s},\;\Delta P_{r,2}^{s}=P_{r_{1},2}^{s}-P_{r_{2},2}^{s}$; $bias_{r,\mathrm{MW}}$ and $bias_{r,P_{IFGF}}$ are receiver-specific bias of $\Delta {\mathrm{MW}}_{r}^{s}$ and $\Delta P_{r,IFGF}^{s}$, respectively. 

As for MW combination, the integer wide-lane ambiguity can be removed by rounding [22]. Then, we can obtain the fractional cycle bias of MW combination. To simplify the expression, the biases of MW combination and pseudorange IFGF combination are rewritten and described as receiver-specific bias and receiver–satellite pair bias:

(4) $$\left\{ \begin{array}{ll} c\cdot bias_{r,\mathrm{MW}}^{s} & =\Delta {\mathrm{MW}}_{r}^{s}-Round(\Delta {\mathrm{MW}}_{r}^{s})\\ & =c\cdot (bias_{type(r),\mathrm{MW}}^{s}+bias_{r,\mathrm{MW}})\\ c\cdot bias_{r,P_{IFGF}}^{s} & =\Delta P_{r,IFGF}^{s}\\ & =c\cdot (bias_{type(r),P_{IFGF}}^{s}+bias_{r,P_{IFGF}}) \end{array}\right.$$

where “Round()” represents rounding operator, $bias_{type(r),P_{IFGF}}^{s}$ and $bias_{type(r),\mathrm{MW}}^{s}$ represent receiver–satellite pair bias of MW and pseudorange IFGF combination, respectively, and the expressions are shown as follows:

(5) $$\left\{ \begin{array}{l} bias_{type(r),\mathrm{MW}}^{s}=\frac{f_{1}\cdot \Delta b_{type(r),1}^{s}+f_{2}\cdot \Delta b_{type(r),2}^{s}}{(f_{1}+f_{2})\cdot \lambda_{1,-1,0}}\\ bias_{type(r),P_{IFGF}}^{s}=(\frac{f_{1}^{2}}{f_{1}^{2}-f_{2}^{2}}-\frac{f_{1}^{2}}{f_{1}^{2}-f_{3}^{2}})\cdot \Delta b_{type(r),1}^{s}-\frac{f_{2}^{2}}{f_{1}^{2}-f_{2}^{2}}\cdot \Delta b_{type(r),2}^{s}+\frac{f_{3}^{2}}{f_{1}^{2}-f_{3}^{2}}\cdot \Delta b_{type(r),3}^{s} \end{array}\right..$$

For each receiver type, based on the biases calculated by different stations, the receiver-specific and receiver–satellite pair biases can be separated as:

(6) $$\left(\begin{array}{c} bias_{1}^{s}\\ bias_{2}^{s}\\ \vdots \\ bias_{n}^{s} \end{array}\right)=\left(\begin{array}{cc} U_{n}⊗u_{m} & u_{n}⊗U_{m} \end{array}\right)\left(\begin{array}{c} bias_{r}\\ bias_{type(r)}^{s} \end{array}\right)$$

in which *n* is the number of stations that are equipped with the same receiver type, ${\mathrm{bias}}_{r}^{s}={\left(\begin{array}{ccc} {\mathrm{bias}}_{r,\;\mathrm{MW}}^{1} & \cdots & {\mathrm{bias}}_{r,\;\mathrm{MW}}^{m} \end{array}\right)}^{T}$ or ${\mathrm{bias}}_{r}^{s}={\left(\begin{array}{ccc} {\mathrm{bias}}_{r,\;P_{IFGF}}^{1} & \cdots & {\mathrm{bias}}_{r,\;P_{IFGF}}^{m} \end{array}\right)}^{T}$ is the vector of pseudorange bias combinations for receiver *r* (*r* = 1, 2, …, *n*); ${\mathrm{bias}}_{r}={\left(\begin{array}{ccc} {\mathrm{bias}}_{1,\;\mathrm{MW}} & \cdots & {\mathrm{bias}}_{n,\;\mathrm{MW}} \end{array}\right)}^{T}$ or ${\mathrm{bias}}_{r}={\left(\begin{array}{ccc} {\mathrm{bias}}_{1,\;P_{IFGF}} & \cdots & {\mathrm{bias}}_{n,\;P_{IFGF}} \end{array}\right)}^{T}$ is the receiver-specific bias vector for the stations used in pseudorange bias estimation; $bias_{type\left(r\right)}^{s}={\left(\begin{array}{ccc} {\mathrm{bias}}_{\mathrm{type}(r),\mathrm{MW}}^{1} & \cdots & b_{\mathrm{type}(r),\mathrm{MW}}^{m} \end{array}\right)}^{T}$ or $bias_{type\left(r\right)}^{s}={\left(\begin{array}{ccc} {\mathrm{bias}}_{\mathrm{type}(r),P_{IFGF}}^{1} & \cdots & {\mathrm{bias}}_{\mathrm{type}(r),P_{IFGF}}^{m} \end{array}\right)}^{T}$ is the receiver-related pseudorange bias for a specific receiver type; $U_{k}$ is the k×k identity matrix where *k* denotes the dimension of the matrix; $u_{k}$ is a k×1 vector with all elements equal to one; ⊗ is the Kronecker product [23]. Since receiver-specific and receiver–satellite pair biases are linearly dependent, in order to eliminate the rank deficiency and solve Equation (6), an extra conditional equation should be introduced as follows:

(7) $$0=\sum_{s=1}^{m}bias_{type(r)}^{s}.$$

Once the receiver-related pseudorange biases of MW combination and pseudorange IFGF combination are determined, by combining the IF code bias corrections, the hardware delay of raw observations can be solved by the following linear equations:

(8) $$\left\{ \begin{array}{l} bias_{type(r),\mathrm{MW}}^{s}=\frac{f_{1}\cdot \Delta b_{type(r),1}^{s}+f_{2}\cdot \Delta b_{type(r),2}^{s}}{(f_{1}+f_{2})\cdot \lambda_{1,-1,0}}\\ bias_{type(r),P_{IFGF}}^{s}=(\frac{f_{1}^{2}}{f_{1}^{2}-f_{2}^{2}}-\frac{f_{1}^{2}}{f_{1}^{2}-f_{3}^{2}})\cdot \Delta b_{type(r),1}^{s}-\frac{f_{2}^{2}}{f_{1}^{2}-f_{2}^{2}}\cdot \Delta b_{type(r),2}^{s}+\frac{f_{3}^{2}}{f_{1}^{2}-f_{3}^{2}}\cdot \Delta b_{type(r),3}^{s}\\ bias_{type(r),P_{IF}}^{s}=\frac{f_{1}^{2}}{f_{1}^{2}-f_{2}^{2}}\cdot \Delta b_{type(r),1}^{s}-\frac{f_{2}^{2}}{f_{1}^{2}-f_{2}^{2}}\cdot \Delta b_{type(r),2}^{s} \end{array}\right.$$

where $bias_{type(r),P_{IF}}^{s}$ is the B1/B2 pseudorange IF combination bias provided by reference [13].

## 3. BDS Pseudorange Bias Calibration

In this section, observations from several GNSS networks are collected for BDS pseudorange bias calibration. With the observation model introduced in the above section, the receiver-related pseudorange biases (${\mathrm{bias}}_{r,\;\mathrm{MW}}^{s}$ and ${\mathrm{bias}}_{r,\;P_{IFGF}}^{s}$) of each receiver type are calculated and analyzed. Based on the characteristic analysis of BDS pseudorange biases, the triple-frequency pseudorange bias corrections for different types of receivers are presented.

### 3.1. Data Collection

**Figure 1:**
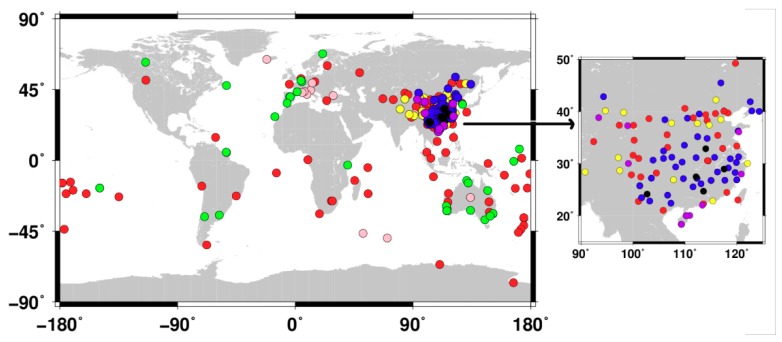
Distribution of stations used for triple-frequency BeiDou Navigation Satellite System (BDS) code bias calibration. TRIMBLE (red circles), LEICA (pink circles), SEPTENTRIO (green circles), UNICORE (yellow circles), COMNAV (blue circles), HI-TARGET (black circles), CHC (purple circles).

Nowadays, there are abundant GNSS stations, which are able to track triple-frequency BDS signals. In this paper, GNSS data, which were collected from the multi-GNSS experiment (MGEX), crustal movement observation network of China [24], the National BDS Augmentation Service System [25], Curtin GNSS CORS, and Hong Kong SatRef GPS Network stations, were used for BDS triple-frequency receiver-related code bias calibration and validation. These stations are globally distributed and equipped with receivers from seven manufacturers. The distribution of the stations is presented in Figure 1.

### 3.2. BDS Pseudorange Bias Analysis and Calibration

Based on the method introduced in Section 2, BDS receiver-related pseudorange biases including ${\mathrm{bias}}_{r,\;\mathrm{MW}}^{s}$ and ${\mathrm{bias}}_{r,\;P_{IFGF}}^{s}$ of different receiver types can be estimated. The data were collected from Day Of Year (DOY) 154 to 160, 2017 with intervals of 30 s. A minimum elevation limit of 20° was applied in BDS code biases estimation to reduce the impacts of multipath at low elevations. In order to reduce the effect of pseudorange noise, the observations were abandoned when the tracking arc length was less than 2 h for a receiver–satellite pair. Moreover, station YNZD was selected as a reference station to be consistent with the models used in reference [13], which can ensure that the two IFGF pseudorange biases estimated in this paper and the B1/B2 IF code biases were on the same basis. Then, we computed BDS code biases for all the other stations relative to station YNZD for each satellite and each day.

**Figure 2:**
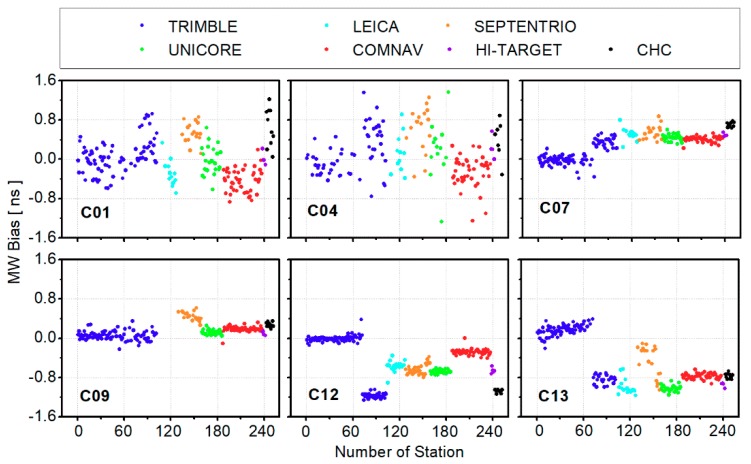
Average BDS receiver-related code biases for different stations (${\mathrm{bias}}_{r,\;\mathrm{MW}}^{s}$) from DOY 154 to 160, 2017; different colors represent different receiver manufacturers.

Figure 2 gives several examples of the BDS receiver-related code biases ${\mathrm{bias}}_{r,\;\mathrm{MW}}^{s}$, including BDS geostationary earth orbit (GEO) satellites C01, C04; inclined geosynchronous orbit (IGSO) satellites C07, C09, C13; and medium earth orbit (MEO) satellite C12. Obviously, the characteristics of the BDS code biases ${\mathrm{bias}}_{r,\;\mathrm{MW}}^{s}$ were similar to that of B1/B2 IF code biases [13]. The BDS code biases generally showed consistence with receiver types. Code biases of C12 were concentrated near −0.7 ns for all COMNAV receivers and about −1.1 ns for all CHC receivers. Moreover, the receiver-related code biases of TRIMBLE NET R9 receivers could be divided into two groups (namely TRIMBLE-1 and TRIMBLE-2). The differences between these two groups of TRIMBLE NET R9 were obvious for C07, C12, and C13, which correspond to about 0.4, −1.2, and −0.9 ns, respectively. In addition, the code bias dispersion of GEO satellites was larger than that of IGSO and MEO satellites due to the heavy multipath for GEO satellites.

**Figure 3:**
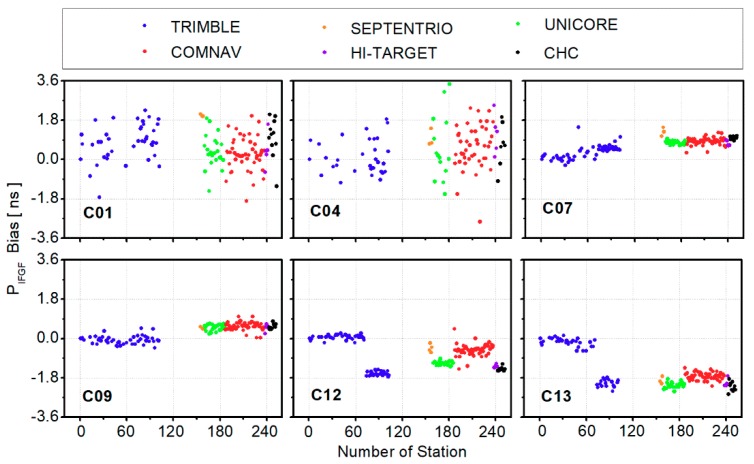
Average BDS receiver-related code biases for different stations (${\mathrm{bias}}_{r,\;P_{IFGF}}^{s}$) from DOY 154 to 160, 2017; different colors represent different receiver manufacturers.

Figure 3 presents the code biases ${\mathrm{bias}}_{r,\;P_{IFGF}}^{s}$ among different stations. Since some receivers were only able to track B1 and B2 signals, the station number used for ${\mathrm{bias}}_{r,\;P_{IFGF}}^{s}$ analysis was less than that used for ${\mathrm{bias}}_{r,\;\mathrm{MW}}^{s}$. Similar to the characteristics of code biases ${\mathrm{bias}}_{r,\;\mathrm{MW}}^{s}$ presented in Figure 2, the code biases ${\mathrm{bias}}_{r,\;P_{IFGF}}^{s}$ were related to receiver types. The maximum difference between different receivers could reach up to about 2.0 ns between TRIMBLE-1 and UNICORE of C13.

**Figure 4:**
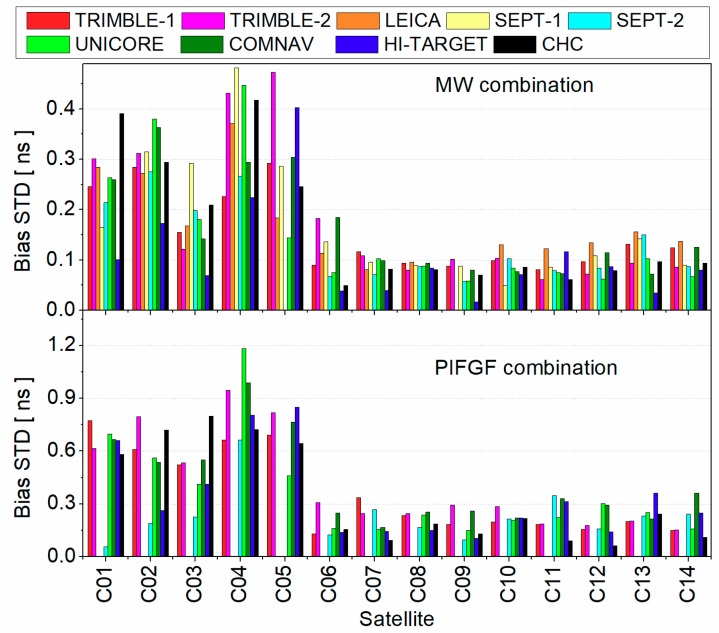
Standard deviation (STD) of pseudorange bias across different stations.

Concerning the consistency of estimated pseudorange bias, Figure 4 presents the standard deviation (STD) among different stations. It is clear that the accuracy of estimated MW and pseudorange IFGF combinations of GEO satellite was worse than that of IGSO and MEO satellites. As for GEO satellites, the average STD of MW combination was about 0.26 ns while it was about 0.53 ns for pseudorange IFGF combination. The bias STD of pseudorange IFGF combination was larger than that of MW combination because of its larger noise amplification factor. As for IGSO and MEO satellites, the average bias STDs were about 0.09 and 0.17 ns for MW and pseudorange combinations, respectively.

**Figure 5:**
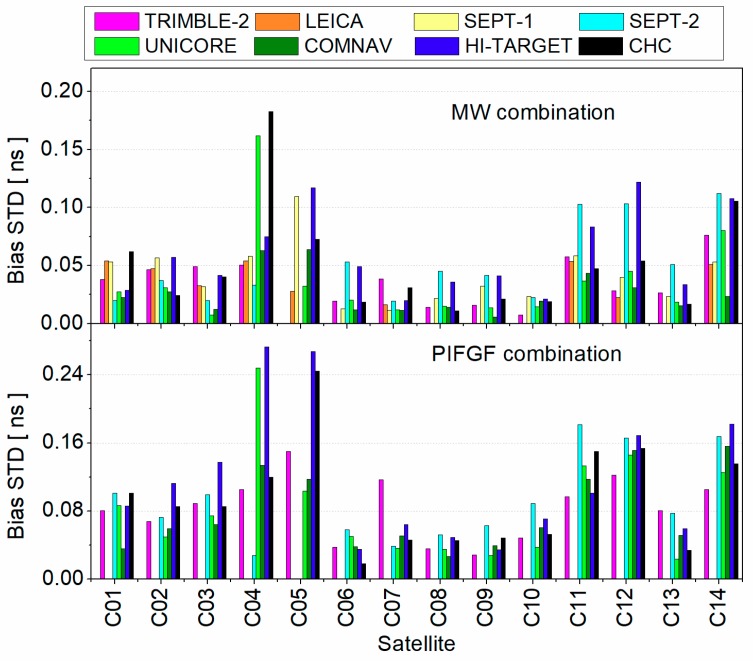
Stability of pseudorange bias across different days.

According to the analysis [13], there is no obvious rule for TRIMBLE NET R9 receivers and it should be tested which groups it belongs to when a TRIMBLE NET R9 receiver is used. Since they are unable to calibrate absolute code biases, receivers of TRIMBLE-1 were chosen as reference, which means corrections of TRIMBLE-1 were zero for all satellites. Figure 5 gives the STD of pseudorange biases across seven -days. Generally, the estimated biases were quite stable over seven days, so that the average STD was about 0.04 and 0.08 ns for MW and pseudorange IFGF combinations, respectively. In particular, the stability of the estimated bias of C04 and C05 was worse than that of other satellites for receivers of UNICORE, COMNAV, HI-TARGET, and CHC. This may be because stations equipped with these receivers were all located in China and C04 and C05 satellites were located in the westernmost and easternmost parts of China (low elevation angle). Furthermore, the estimated bias stability of MEO satellites was lower than that of IGSO satellites. The reason may be that the orbital period of BDS IGSO satellite is one day and that of BDS MEO satellite is seven days. Thus, some unmodeled error will decrease the accuracy of estimated MEO pseudorange bias [12,26].

**Figure 6:**
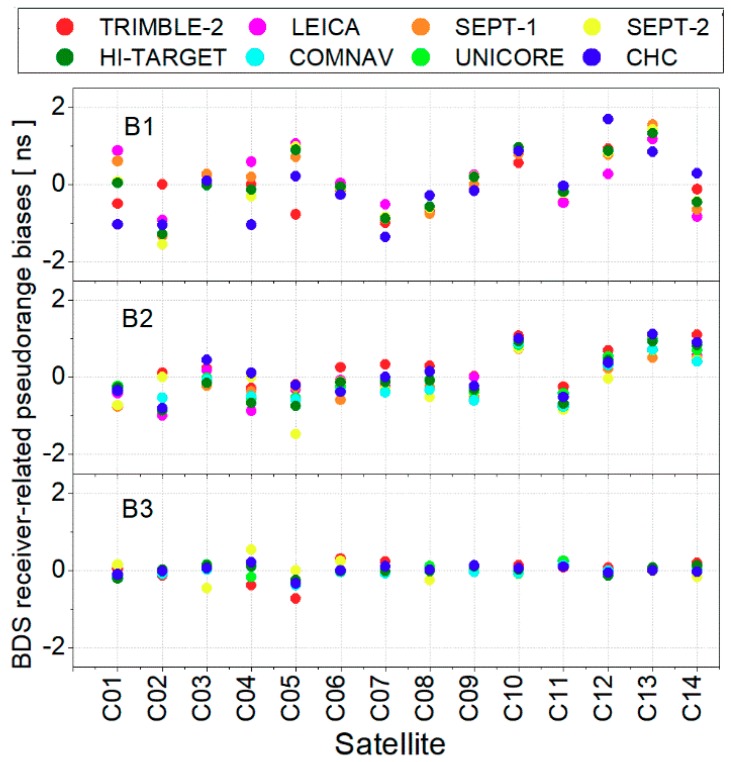
Value of BDS triple-frequency receiver-related pseudorange biases.

By combining the receiver-related code biases ${\mathrm{bias}}_{r,\;\mathrm{MW}}^{s}$, ${\mathrm{bias}}_{r,\;P_{IFGF}}^{s}$ calculated in this study and the B1/B2 IF code biases, sets of corrections for BDS raw pseudorange observations of different receiver types were computed based on Equation (8) and are presented in Figure 6. In addition, to remove the BDS receiver-related code biases, BDS code bias corrections provided in this paper should be added to pseudorange observations. Meanwhile, all the corrections can be downloaded from https://www.researchgate.net/project/GNSS-Biases/update/5cd2cff83843b0b98251d8ed. Anyone can directly correct these biases to improve BDS data processing accuracy by any software.

## 4. Validation by BDS Precise Positioning and Ambiguity Resolution

**Figure 7:**
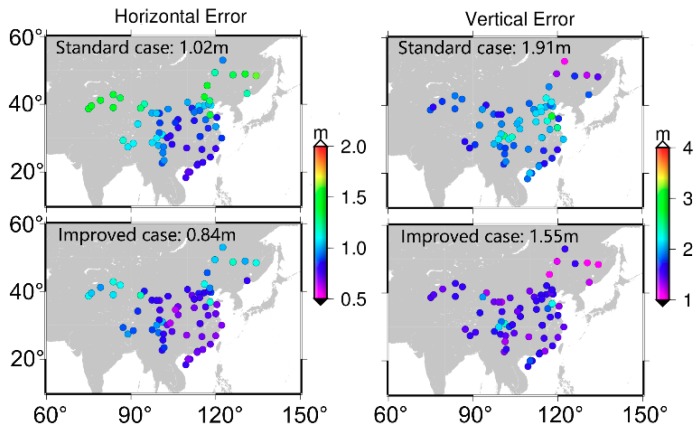
Single-frequency single point positioning (SF-SPP) average positioning accuracy by using B1 signal (averaged Root Mean Square (RMS), DOY 175–181, 2017); top two panels are results of the standard case; bottom two panels are results of the improved case.

As discussed above, it was seen that BDS code biases were relevant to receiver types. In this section, to validate the code bias corrections proposed in this paper, several experiments including BDS single-frequency single point positioning (SF-SPP), single-frequency PPP (SF-PPP), dual-frequency PPP (DF-PPP) and wide-lane UPD estimation and ambiguity resolution at stations equipped with different types of receivers are carried out. A total of 70 stations with one-week observations from DOY 175 to 181, 2017 are used in this section. All these stations are located in China and their distributions are presented in Figure 7. These stations are equipped with TRIMBLE-1, UNICORE, COMNAV, HI-TARGET, and CHC receivers.

### 4.1. Processing Strategy

In the processing of SPP, to achieve the highest positioning accuracy, users should implement the same conventions and models as those adopted by the International GNSS Service (IGS) products. Since BDS receiver-related code biases are ignored in the estimation of BDS satellite clock and DCB provided by GeoForschungsZentrum (GFZ) and Deutschen Zentrums für Luft- und Raumfahrt (DLR), these products should be recalculated with the consideration of BDS receiver-related code biases when evaluating the performances of positioning. Based on these newly estimated products, different positioning modes were carried out (named as the improved case). As a comparison, positioning results by using the satellite clock from GFZ and MGEX DLR DCB were also employed (named as the standard case). The details of the positioning model are listed in Table 1.

### 4.2. Accuracy of BDS Single-Frequency SPP

In this section, BDS SF-SPP by using B1, B2, and B3 signals is conducted to validate the corrections proposed. Figure 7 presents the distribution of 70 stations and different colors represent the accuracy of SF-SPP using B1 signal. It is obvious that, compared to the standard case, positioning accuracy of the improved case was greatly improved. The average positioning accuracy was improved from 1.02 and 1.91 m to 0.84 and 1.55 m in horizontal and vertical components, respectively. Moreover, the positioning performance of different stations showed obvious geographical features since BDS can only provide regional navigation and positioning services at present. For the horizontal component, the accuracy in northeast and northwest China was worse than that in other areas for both the standard and improved cases. However, it showed a different characteristic for the vertical component. The accuracy of vertical component in the central region of China was worse than that in the other areas, especially for the standard case.

**Figure 8:**
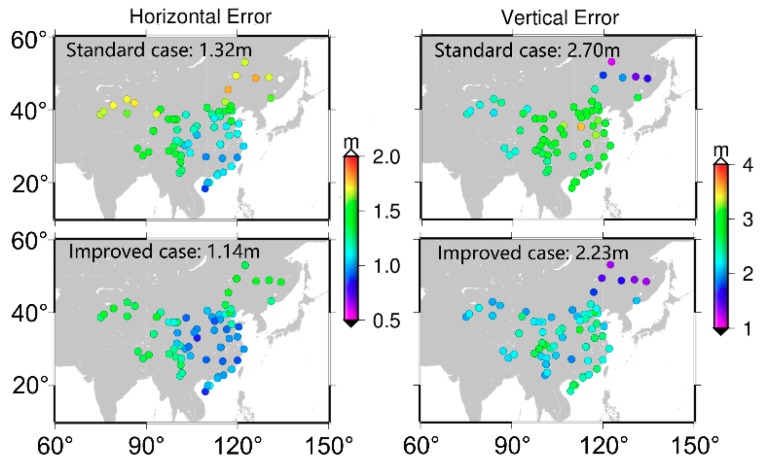
SF-SPP average positioning accuracy by using B2 signal (averaged RMS, DOY 175–181, 2017); top two panels are results of the standard case; bottom two panels are results of the improved case.

**Figure 9:**
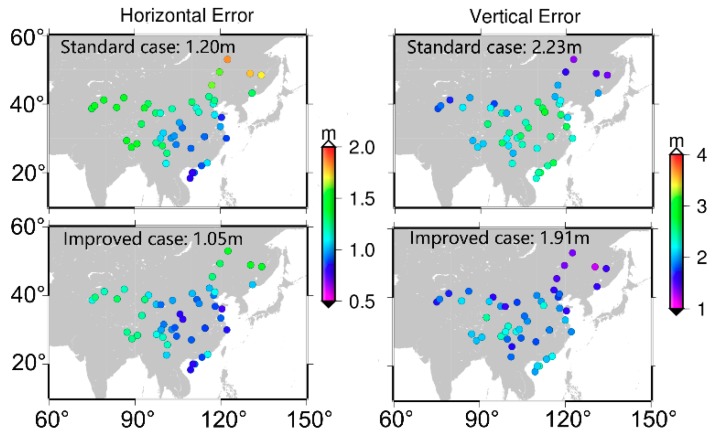
SF-SPP average positioning accuracy by using B3 signal (averaged RMS, DOY 175–181, 2017); top two panels are results of the standard case; bottom two panels are results of the improved case.

Figure 8 and Figure 9 are the positioning results of SF-SPP using B2 and B3 signals, respectively. Similar to the above analysis of Figure 7, the accuracy of both horizontal and vertical components was significantly improved when the BDS code biases were corrected. The average positioning accuracy was improved from 1.32 and 2.70 m to 1.14 and 2.23 m for SF-SPP by using B2 signal in horizontal and vertical components, respectively. As for SF-SPP by using B3 signal, the average accuracy was improved from 1.20 and 2.23 m to 1.05 and 1.91 m in horizontal and vertical components, respectively. Similarly, the distribution of positioning accuracy showed the same regular pattern as in Figure 7. Moreover, among B1, B2, and B3 SF-SPP solutions, positioning accuracy by using the B2 signal was worse than positioning accuracy by using B1 and B3. The station number used for B3 SF-SPP was less than that for B1 and B2 SF-SPP because some stations cannot track B3 signal.

### 4.3. Convergence Time of BDS Precise Point Positioning

In this section, the convergence performances of SF-PPP by using B1, B2, and B3 signals and DF-PPP by using B1 and B2 ionosphere-free combination are analyzed and compared. In order to evaluate the performance of convergence time, the SRIF filter of SF-PPP and DF-PPP at each station was reset every 4 h for both the standard case and the improved case. All the stations from DOY 175 to 181, 2017 used in the evaluation of positioning performance are processed in this section. A total of 2940 independent PPP results were evaluated for convergence analysis.

**Figure 10:**
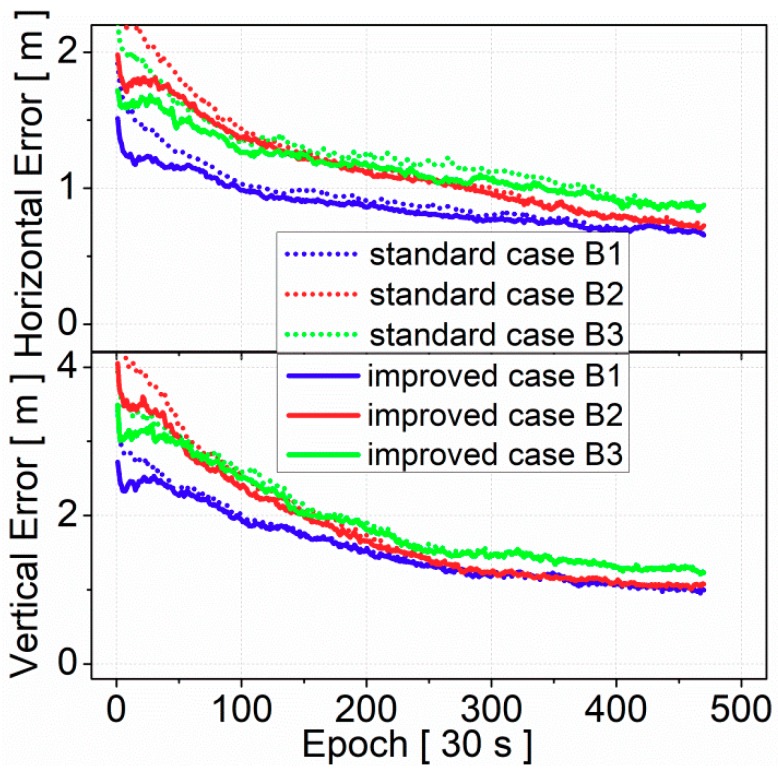
Convergence performance of SF-PPP at the 95% level (statistics from 70 stations, DOY 175–181, 2017); solid lines and dots represent the standard case and the improved case, respectively.

Figure 10 presents the convergence time of BDS SF-PPP by using B1, B2, and B3 signals at the 95% level. Compared to the standard case, the results of the improved case proved that convergence time was shortened in both horizontal and vertical components. The improvements of positioning accuracy were more significant at the beginning. As for SF-PPP by B1 signal, compared to the standard case, the positioning accuracy of the improved case was improved from 1.48 and 2.75 m to 1.22 and 2.49 m at 10 min initial time in horizontal and vertical components, respectively. However, the positioning accuracy was only improved from 0.99 and 1.88 m to 0.92 and 1.83 m at 1 h in horizontal and vertical components, respectively. Furthermore, the positioning accuracy generally showed no difference between the standard case and the improved case after 3 h convergence. This is quite reasonable since the BDS receiver-related code biases only introduce biases in the pseudorange observations, which can be absorbed by ambiguity in PPP processing, therefore, the positioning accuracy will not be lost after the PPP is initialized.

**Figure 11:**
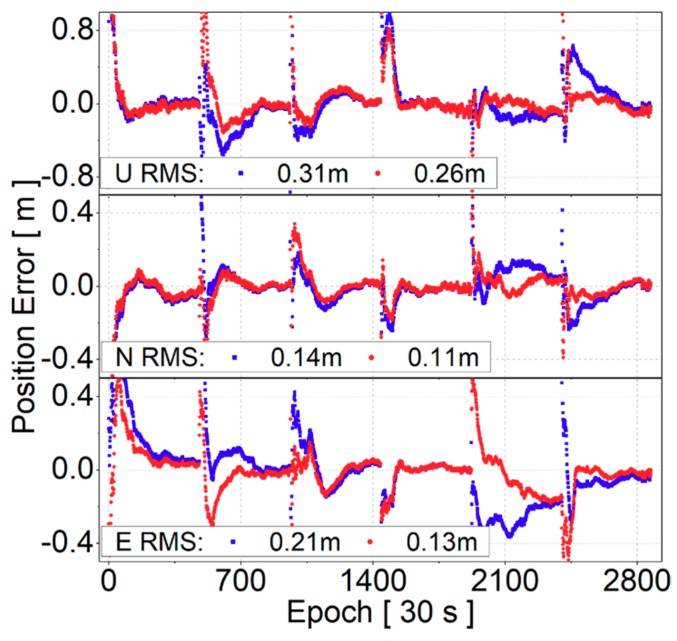
Dual-frequency precise point positioning (DF-PPP) positioning error time series at station CHHC, DOY 180, 2017; blue points are results of the standard case and red points are results of the improved case.

Figure 11 plots the positioning time series of DF-PPP at station CHHC. The result demonstrates that it was able to reach centimeter level positioning accuracy for both the cases after convergence. Moreover, it shows that positioning accuracy was generally improved in up/north/east components when the BDS receiver-related code bias corrections were employed. The RMS improvements in up/north/east components were from 0.31, 0.14, and 0.21 m to 0.26, 0.11, and 0.13 m, which correspond to 16.1%, 21.4%, and 38.1%, respectively. It should be pointed out that the RMS improvement should be attributed to a faster convergence when the code biases are corrected.

**Figure 12:**
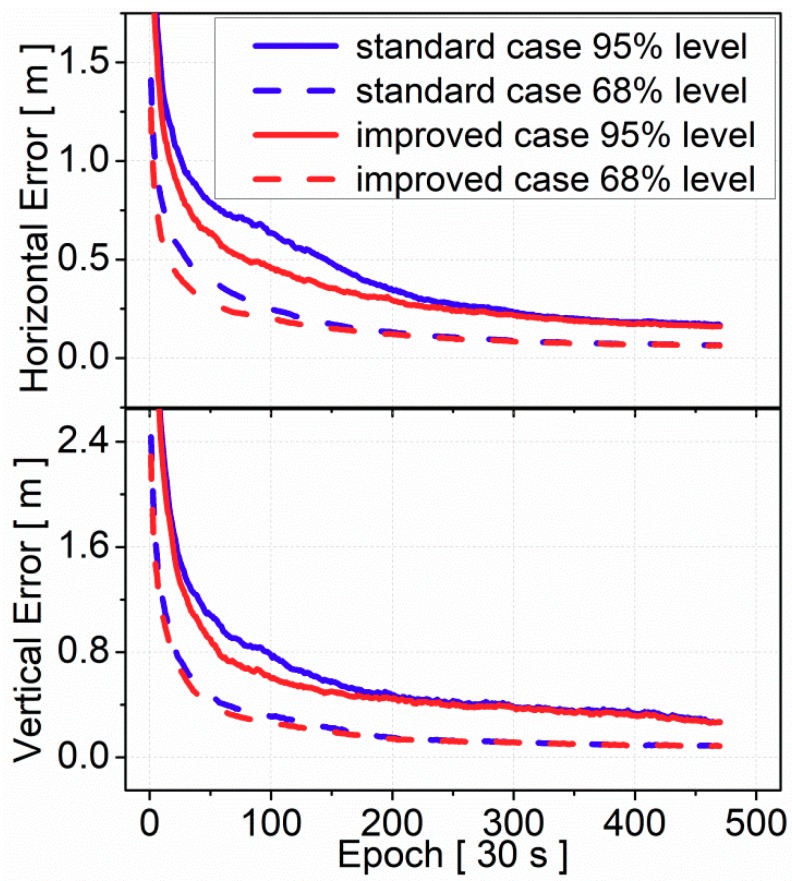
Convergence performance of DF-PPP at the 95% and 68% level (statistics from 70 stations, DOY 175–181, 2017); blue lines are results of the standard case and red lines are results of the improved case; solid lines are results at the 95% level and dash lines are results at the 68% level.

Figure 12 represents positioning error at the 95% and 68% level as a function of time since the PPP start. A total of 2940 independent PPP results were evaluated for DF-PPP convergence analysis. Compared to the standard case, the results of the improved case also proved that convergence time was shortened in both horizontal and vertical components. It took 150 epochs (about 1.2 h) for the standard case to reach 0.5 m in horizontal component at the 95% level while it only took 75 epochs (about 0.6 h) to reach the same accuracy for the improved case. Similar to the results of SF-PPP, the DF-PPP positioning accuracy after convergence was at the same level for the standard case and the improved case. 

Comparing the SF-SPP, SF-PPP, and DF-PPP performance in terms of positioning accuracy and convergence time proves the effectiveness of code bias corrections in BDS precise positioning. It should be pointed out that the code biases must be corrected at the same time in the estimation of satellite clock and DCB and user terminal. Otherwise, the effect of the code bias corrections will be not significant.

### 4.4. Wide-Lane UPD Estimation and Ambiguity Resolution

**Figure 13:**
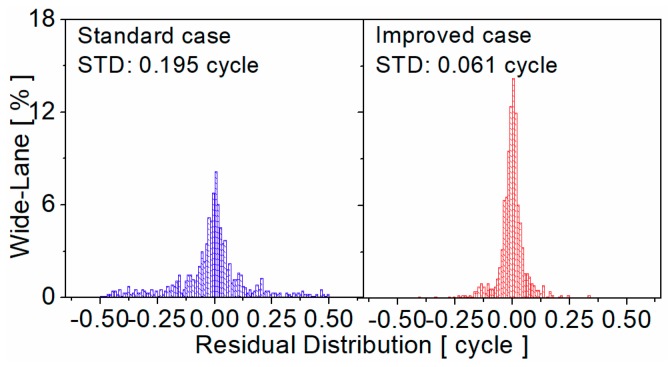
Residual distribution of wide-lane UPD for standard case (**left panel**) and improved case (**right panel**).

The BDS receiver-related pseudorange bias will greatly influence the effectiveness of wide-lane ambiguity resolution based on MW combination. In this section, wide-lane UPD estimation and ambiguity resolution are evaluated and compared for the standard case (without bias correction) and the improved case (with bias correction). The UPD consistency among float ambiguities of different stations plays an important role in un-difference ambiguity resolution and can reflect the quality of UPD estimation. Figure 13 presents the residual distribution of wide-lane UPD among different stations. Obviously, the residuals performed better when receiver-related pseudorange bias corrections were used. For the standard case, the residuals distributed between ±0.5 cycles. However, most of the residuals were concentrated between ±0.25 cycles for the improved case, which validates the reliability of receiver-related pseudorange bias corrections. Finally, the STD of wide-lane UPD residuals are 0.195 and 0.061 cycles for the standard case and improved case, respectively.

Based on the UPDs estimated, the un-difference wide-lane ambiguity resolution was performed. The fixing decision was made according to the probability [30]. Then, the success rate of ambiguity resolution is counted as follow:

(9) $$P_{suc}=\frac{n_{fix}}{n_{all}}\cdot 100\%$$

where *n_fix_* is the number of fixed ambiguities; *n_all_* is the number of all candidate ambiguities.

**Figure 14:**
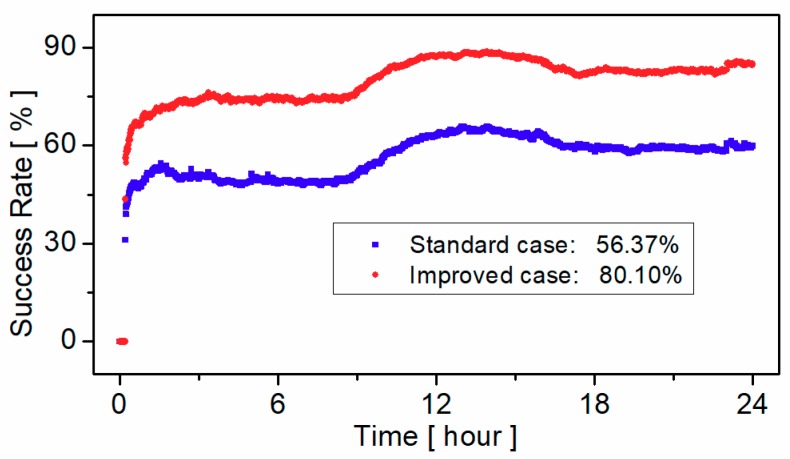
Success rate of wide-lane ambiguity resolution without correction (blue points) and with correction (red points).

Figure 14 presents the seven-day average success rate of wide-lane ambiguity resolution. Since the ambiguity resolution is processed in simulated real-time mode, the success rate is generally lower than that in post-processed mode. As for the standard case, the average success rate of wide-lane ambiguity resolution was 56.37%. Compared to the standard case, the success rate of wide-lane ambiguity resolution of the improved case was significantly improved and could reach up to 80.10%. The improvement was 42.10%.

## 5. Conclusions and Outlook

This study is the continuing work of BDS receiver-related code biases [12]. It is observed that receiver-related code biases exist in BDS pseudorange measurements, which can reach up to about 3 ns for B1/B2 IF pseudorange observations among different types of receivers. The IF combination of the B1 and B2 receiver-related code biases are calibrated for different receivers from seven receiver manufactures. However, the correction model can only be suitable for B1 and B2 dual-frequency IF data processing and is not applicable in BDS single-frequency or triple-frequency data processing. In this contribution, by introducing two additional IFGF combinations, we proposed a method to estimate the BDS triple-frequency receiver-related code biases using network observations from 257 stations. The corrections of BDS receiver-related code biases of B1, B2, and B3 are given for different receiver manufacturers including TRIMBLE, LEICA, SEPTENTRIO, UNICORE, COMNAV, HI-TARGET, and CHC.

In order to validate the availability and effectiveness of the BDS code bias corrections, precise positioning with different frequency observations from different receiver types are processed with and without code bias corrections, including SF-SPP, SF-PPP, DF-PPP, wide-lane UPD estimation, and ambiguity resolution. The SPP performance in different regions reveals that the positioning accuracy is not overall uniform as BDS-2 is still a regional navigation system, the geometric distribution of satellite is poorer in the northeastern and northwestern regions, and correspondingly the positioning accuracy will be worse. By correcting the receiver-related code biases, the positioning accuracy of SPP is improved about 20% on average. The positioning accuracy of single- and dual-frequency PPP at the initial time are also improved. It means the receiver-related code bias corrections play an important role in BDS precise positioning. Moreover, the consistency of wide-lane UPD is greatly improved and the success rate of wide-lane ambiguity resolution is improved 42.10% by correcting receiver-related pseudorange biases. It means the receiver-related code bias corrections play an important role in BDS precise data processing. Thus, experiments should be carried out to analyze the characteristics of pseudorange observations of other navigation systems in the future since receiver-related code bias will greatly affect GNSS data processing.

The research has shown that the pseudorange biases of different BDS satellites at the receiver are not the same. Therefore, it is impractical to estimate one receiver DCB per station during ionospheric modeling and DCB estimation with BDS observations. It should be recalculated and compared with the DCB products from MGEX. Furthermore, in the future, the classification of different receivers into consistent groups for different satellite navigation systems should be further studied, which is of great importance in multi-GNSS precise data processing. Since the IGS bias working group has published SINEX_BIAS V1.00, which supports bias correction according to receiver group, IGS analysis centers should consider providing BDS receiver-related code bias corrections in their products with SINEX_BIAS format.

## Figures and Tables

**Table 1:** Details of precise positioning.

Ionospheric Delay	Corrected by Global Ionospheric Map Products Provided by the Center of Orbit Determination in Europe
Sample rate (s)	30
Cutoff elevation (°)	7
Tropospheric delay	Saastamoinen model with GMF mapping functions [27,28]
Satellite phase center	Only phase center offset (PCO) correction used by GFZ
Satellite orbit and clock	Products provided by GFZ for the standard case recalculated for the improved case [13]
DCB	Products provided by DLR for the standard case recalculated for the improved case [9]
Filter	Square root information filter [29]

GMF—global mapping function; GFZ—GeoForschungsZentrum; DCB—differential code bias; DLR—Deutschen Zentrums für Luft- und Raumfahrt.
